# Reconstitution and structure of a bacterial Pnkp1–Rnl–Hen1 RNA repair complex

**DOI:** 10.1038/ncomms7876

**Published:** 2015-04-17

**Authors:** Pei Wang, Kiruthika Selvadurai, Raven H. Huang

**Affiliations:** 1 Department of Biochemistry, University of Illinois at Urbana-Champaign, 600 South Mathews Avenue, Urbana, Illinois 61801, USA

## Abstract

Ribotoxins cleave essential RNAs for cell killing, and RNA repair neutralizes the damage inflicted by ribotoxins for cell survival. Here we report a new bacterial RNA repair complex that performs RNA repair linked to immunity. This new RNA repair complex is a 270-kDa heterohexamer composed of three proteins—Pnkp1, Rnl and Hen1—that are required to repair ribotoxin-cleaved RNA *in vitro*. The crystal structure of the complex reveals the molecular architecture of the heterohexamer as two rhomboid-shaped ring structures of Pnkp1–Rnl–Hen1 heterotrimer fused at the Pnkp1 dimer interface. The four active sites required for RNA repair are located on the inner rim of each ring. The architecture and the locations of the active sites of the Pnkp1–Rnl–Hen1 heterohexamer suggest an ordered series of repair reactions at the broken RNA ends that confer immunity to recurrent damage.

## Abstract (web summary)

Bacterial ribotoxins target and cleave RNAs involved in translation, with target organism resistance being mediated by RNA repair systems. Here, Wang *et al.* identify, report the crystal structure, and characterize the activity of Pnkp1–Rnl–Hen1, a novel RNA repair complex from *Capnocytophaga gingivalis*.

## Graphical abstract

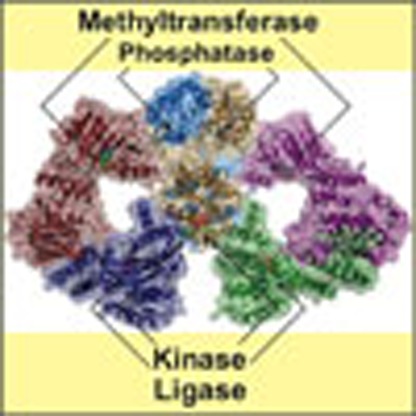

Competition between organisms, microbes in particular, for a limited resource of nutrition require adaptation at many levels; some organisms employ a variety of toxins to eliminate competition, while others use molecular defence systems to neutralize toxins for survival. Among many cellular targets of toxins, the protein translation apparatus is among the more frequently targeted systems due to its conservation and essential role in all organisms. A majority of toxins targeting protein synthesis appear to be ribotoxins, which cleave essential RNAs (ribosomal RNA, transfer RNA (tRNA) and messenger RNA) involved in protein translation. With the exception of several ribotoxins generated in eukaryotic organisms^1^,^2^,^3^,^4^,^5^, most ribotoxins seem to be produced by prokaryotes, found in toxin-antitoxin^6^, abortive infection^7^ and polymorphic toxin systems^8^. Bioinformatic analysis of 2,181 prokaryotic genomes revealed an estimated ∼10,000 toxins belonging to toxin-antitoxin systems^9^, and a similar number of secreted toxins were predicted in the form of polymorphic toxins^8^. These reasons suggest that ribotoxins might constitute the biggest percentage of toxins in living organisms^10^,^11^.

With the exception of ricin and some metal-dependent ribotoxins^2^,^12^, most ribotoxins are metal independent and employ a transesterification mechanism to cleave RNAs, generating a hydroxyl group at the 5′ end and a 2′,3′-cyclic phosphate group at the 3′ end^13^,^14^,^15^,^16^,^17^,^18^,^19^ (Supplementary Fig. 1). To counter the damage inflicted by ribotoxins, organisms employ protein enzymes to repair the damaged RNA, exemplified by the first RNA repair system from bacteriophage T4 discovered ∼30 years ago^20^ (Supplementary Fig. 2a). The T4 RNA repair system is composed of two proteins named Pnkp and Rnl1, with the former processing the two ends of the damaged RNA and the latter ligating the two processed ends to restore the damaged RNA to its original form (Supplementary Fig. 1).

Building on the study by Martin and Shuman^21^, we discovered a bacterial RNA repair complex comprised of two proteins named Pnkp and Hen1, which form a heterotetramer *in vitro*^22^ (Supplementary Fig. 2b). In addition to the three enzymatic activities also seen in the T4 RNA repair system, the Pnkp–Hen1 RNA repair complex possesses a fourth enzymatic activity in Hen1, which carries out methylation of the 2′-OH group that is responsible for the RNA cleavage (Supplementary Fig. 1). Due to 2′-*O*-methylation, the RNA repaired by Pnkp–Hen1 resists future RNA damage by the same ribotoxin. Thus, the bacterial Pnkp–Hen1 complex performs RNA repair with immunity.

Because methylated RNA repair is superior, there is great incentive for the Pnkp–Hen1 repair complex to have evolved to maximize the production of methylated RNA repair products. In theory, this can be achieved if the RNA repair complex adopts a molecular arrangement and architecture that (i) does not permit RNA repair to occur without Hen1, and (ii) is conducive for efficient 2′-*O*-methylation during RNA repair. Recent studies carried out in the Shuman laboratory as well as ours revealed the molecular mechanism for achieving the first condition^23^,^24^. Specifically, the study by Shuman *et al.*^23^ found that the ligase domain of Pnkp is disabled by a flexible insertion domain that blocks the ligase active site. Our study revealed that the N-terminal domain of Hen1 activates the ligase activity of Pnkp by locking the insertion domain of Pnkp into a conformation that allows access to the ligase pocket^24^. Such an arrangement for Hen1-dependent RNA repair guarantees Hen1 the opportunity to carry out 2′-*O*-methylation during RNA repair.

Addressing the second condition requires knowledge of relative locations of the active sites of phosphatase, methyltransferase and ligase, all of which must be traversed by the 3′ end of the damaged RNA in that particular order to produce the methylated RNA repair product (Supplementary Fig. 1). A structure of the Pnkp–Hen1 heterotetramer would provide such an insight. Despite extensive effort, we were not able to produce diffraction quality crystals of the Pnkp1–Hen1 heterotetramer. However, we were successful in performing structural study of a newly discovered bacterial RNA repair complex consisting of three proteins named Pnkp1, Rnl and Hen1 (Supplementary Fig. 2c), which form a heterohexamer *in vitro*. The new RNA repair complex is distinct from the Pnkp–Hen1 RNA repair complex, but also shares several features (Supplementary Fig. 2c, and compare with Supplementary Fig. 2b). The structure of the new RNA repair complex reported here provides molecular insight into efficient 2′-*O*-methylation during RNA repair. Because of their similarities, this principle may hold true for the Pnkp–Hen1 RNA repair complex. Here we describe the discovery, followed by biochemical and structural characterization of the new Pnkp1–Rnl–Hen1 RNA repair complex.

## Results

### Discovery of a new RNA repair complex in bacteria

The Pnkp–Hen1 RNA repair complex is found in ∼250 bacterial species. Both proteins are highly conserved and exhibit ∼40–60% sequence identities. To investigate whether distant Hen1 is present in bacteria, we performed a comprehensive BLASTP search using the sequence of *Ava*Hen1. We discovered 10 bacterial species with a gene encoding Hen1 that is modestly homologous (∼20%) to *Ava*Hen1. However, unlike the genes encoding Pnkp and Hen1, which are always present in the same operon (Supplementary Fig. 3a), a gene encoding Pnkp homologue could not be found near *hen1* in these organisms. Instead, an operon that is far away from *hen1* was found to contain two genes encoding proteins that may be involved in RNA repair (Supplementary Fig. 3b). One encodes a protein that is equivalent to the ligase domain of bacterial Pnkp with modest sequence identities (∼20%) (Supplementary Fig. 2, and compare Supplementary Fig. 2c with Supplementary Fig. 2b), and the other encodes a protein homologous (∼30% identities) to *T4*Pnkp (Supplementary Fig. 2, and compare Supplementary Fig. 2c with Supplementary Fig. 2a). Because these two proteins are functionally equivalent to Pnkp in the bacterial Pnkp–Hen1 RNA repair complex, we hypothesized that these two proteins, together with the newly found Hen1, may constitute a new RNA repair complex in these 10 bacterial species (Supplementary Fig. 2c). Since the newly found RNA repair system possesses elements of both T4 and Pnkp–Hen1 RNA repair systems, it can be regarded as a hybrid RNA repair system. To distinguish the proteins of the newly discovered RNA repair system from the previously characterized Pnkp–Hen1 system, we tentatively named the ligase homologue as bacterial Rnl (RNA ligase), and the *T4*Pnkp homologue as bacterial Pnkp1 (Supplementary Fig. 2c).

### Reconstitution of the Pnkp1–Rnl–Hen1 RNA repair complex

**Figure 1:**
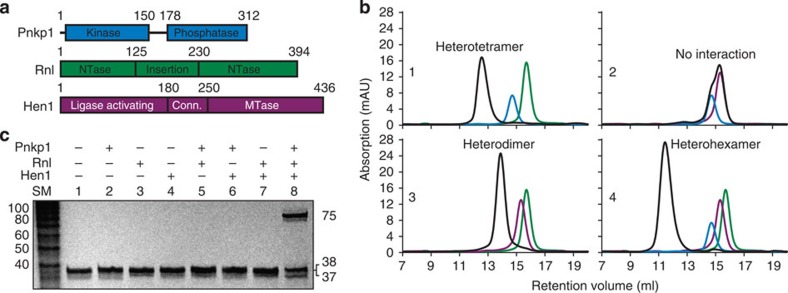
Reconstitution of the Pnkp1–Rnl–Hen1 RNA repair complex *in vitro.* (**a**) Schematic view of the three proteins that constitute the new bacterial RNA repair system. The boundary of domains in each protein was determined based on the structure of the Pnkp1–Rnl–Hen1 heterohexamer. NTase, nucleotidyltransferase domain; Conn., connecting domain; MTase, methyltransferase. (**b**) Size-exclusion chromatography analyses of individual protein, the pairwise mixture and the three-protein mixture. The chromatographic curves of individual proteins are coloured the same as in **a** and the ones for the mixtures are in black. (**c**) Repair assay of a ribotoxin-cleaved tRNA by various combinations of Pnkp1, Rnl and Hen1 as indicated. SM, size marker.

To test the hypothesis that bacterial Pnkp1, Rnl and Hen1 may constitute a new RNA repair system, we cloned the genes encoding Pnkp1, Rnl and Hen1 from *Capnocytophaga gingivalis* (*Cgi*) into an overexpression vector and purified all three recombinant proteins individually (Supplementary Fig. 4). We first carried out chromatographic analyses with the purified recombinant proteins to probe interactions among them (Fig. 1b). Size-exclusion chromatography revealed that Pnkp1 forms a homodimer in solution (Fig. 1b, cyan curve), whereas both Rnl and Hen1 exist as a monomer (Fig. 1b, green and magenta). Analyses of pairwise mixtures in equimolar indicated that Pnkp1 and Rnl form a heterotetramer (Fig. 1b, panel 1), Rnl and Hen1 form a heterodimer (Fig. 1b, panel 3), but Pnkp1 and Hen1 do not interact (Fig. 1b, panel 2). When all three proteins were present in an equimolar mixture, a single species was formed (Fig. 1b, panel 4). Judging by its elution volume, the three proteins form a Pnkp1–Rnl–Hen1 heterohexamer.

To assess RNA repair capability, we employed a ribotoxin-cleaved tRNA as the substrate, which was previously used for *in vitro* reconstitution of the Pnkp–Hen1 RNA repair complex^22^. As shown in Fig. 1c, the damaged tRNA could efficiently be repaired, but only in the presence of all three proteins (Fig. 1c, lane 8). Therefore, the Pnkp1–Rnl–Hen1 heterohexamer is likely the functional unit of a new bacterial RNA repair system *in vivo*. Because the Pnkp1–Rnl–Hen1 RNA heterohexamer possesses two identical copies of each enzymatic activity, a total of eight active sites are present in the complex, same as in the RNA repair complex of the Pnkp–Hen1 heterotetramer.

**Figure 2:**
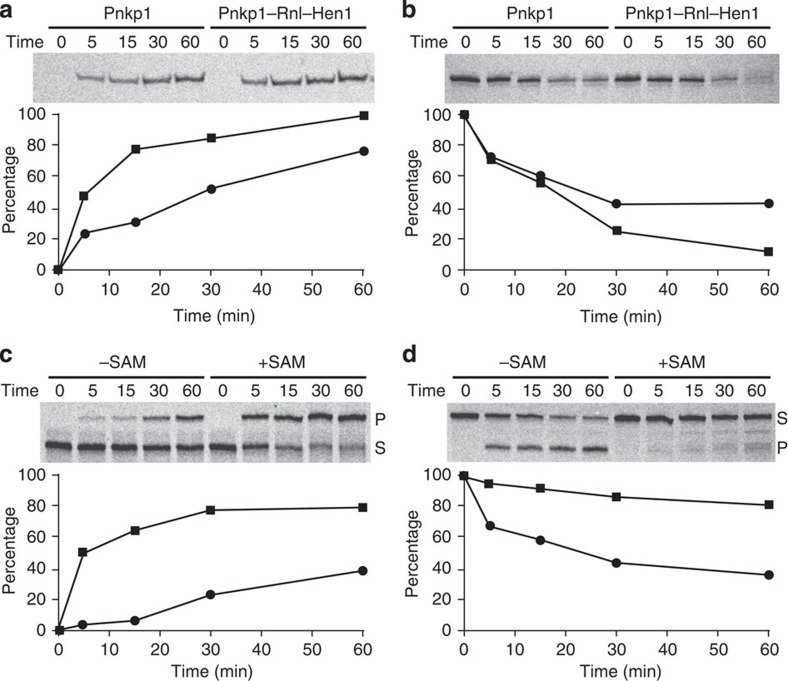
Kinetics of an individual enzymatic reaction. Enzymatic reactions of 5′-phosphoylation (**a**), 3′-dephosphoylation (**b**), RNA ligation (**c**) and RNA cleavage (**d**) were carried out at different time points and quantified, producing the time courses of the reactions. Of each panel of data, the curve marked with a circle represents the results of the left half of the panel, and the one marked with a square denotes the right half. The ratios of substrate to enzyme are 400 for the 5′-phosphorylation reaction shown in **a**, 4 for the 3′-dephosphoylation reaction shown in **b**, 4 for the ligation reaction shown in **c** and 1 for the cleavage reaction shown in **d**. The percentage in **a** represents the maximal value achieved in the experiment, which was estimated to be at least 90% of the 5′-OH RNA ends. The percentage in **c** represents the RNA ligation product formed, and the one in **d** denotes the tRNA that remains uncut. S, substrate; P, product. Representative results are shown.

To evaluate relative rates of four enzymatic activities of the Pnkp1–Rnl–Hen1 heterohexamer, we performed time-dependent reactions focusing on individual enzymatic activity (Fig. 2; Supplementary Figs 5b–d and 6). For these assays, we employed tRNA^Arg^-ΔT, which allowed RNA substrates to be prepared by purifying both 5′- and 3′-half RNA generated by colicin D (cleavage of the full-length tRNA^Arg^ produces two RNA fragments that are 37 and 38 nucleotides in size, which cannot be separated). Cleaved tRNA^Arg^-ΔT has previously been shown to be an effective substrate of RNA repair carried out by the Pnkp–Hen1 heterotetramer^25^. Among the enzymatic reactions we have assayed, 5′-phosphorylation of the 3′-half RNA, carried out by the kinase domain of Pnkp1, is the fastest (Fig. 2a). Formation of the Pnkp1–Rnl–Hen1 heterohexamer has a positive effect on kinase reaction (Fig. 2a, and compare the two curves), but the effect is modest (approximately a twofold rate enhancement). The 3′-dephosphorylation of the 5′-half RNA, performed by the phosphatase domain of Pnkp1, is significantly slower than the kinase reaction (Fig. 2b). On the basis of the difference of substrate to enzyme ratios employed for these two reactions, the rate difference is in the range of 100-fold. As in the case of the kinase reaction, formation of the Pnkp1–Rnl–Hen1 heterohexamer has a positive but modest effect on the dephosphorylation reaction (Fig. 2b, and compare the two curves).

To assess the effect of 2′-*O*-methylation on RNA ligation, the RNA substrates with the processed ends (for example, RNAs with 3'-OH and 5′-PO_4_) were subjected to RNA ligation by the Rnl–Hen1 heterodimer in the absence or presence of *S*-adenosylmethionine (SAM) (Fig. 2c). The presence of SAM significantly increased the rate and efficiency of RNA ligation (Fig. 2c, and compare the two curves). Judged by the initial reaction rates (for example, the data points of the 5-min reactions), the rate enhancement is at least tenfold. When the Pnkp1–Rnl–Hen1 heterohexamer replaced the Rnl–Hen1 heterodimer for the reactions, similar differences of the rate and efficiency of RNA ligations without and with SAM were observed (Supplementary Fig. 6). Surprisingly, the Pnkp1–Rnl–Hen1 heterohexamer ligates cleaved tRNA^Arg^-ΔT at a slower rate than the one carried out by the Rnl–Hen1 heterodimer (compare Supplementary Fig. 6 with Fig. 2c). The mechanisms that lead to these observations described here are unknown and are likely to be complicated, which may require additional investigation that is beyond the scope of this study.

To assess the extent of 2′-*O*-methylation, the RNA repair products shown in Fig. 2c were purified and subjected to cleavage by colicin D (Fig. 2d). The repaired RNA produced in the presence of SAM demonstrated a significantly stronger resistance to the cleavage by colicin D compared with the one produced without SAM (Fig. 2d, and compare the two curves), indicating that 2′-*O*-methylation by Hen1 during RNA repair results in immunity of the repaired RNA to the ribotoxin. On the basis of the difference of cleavage shown in Fig. 2d, ∼50% of the repaired RNA is 2′-*O*-methylated at the junction of repair. The incomplete cleavage of the repaired RNA without 2′-*O*-methylation might be due to partial misfolding of tRNA^Arg^-ΔT (Fig. 2d, the curve marked with cycle).

### Structure of the Pnkp1–Rnl–Hen1 heterohexamer

**Figure 3:**
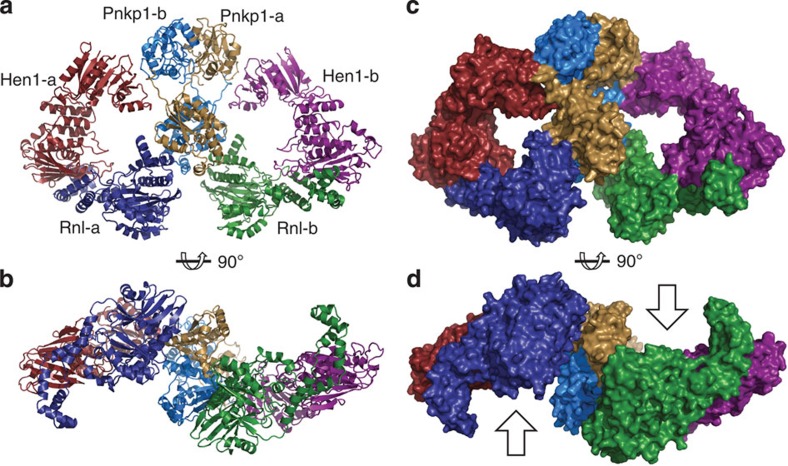
Overall structure of the Pnkp1–Rnl–Hen1 heterohexamer. (**a**,**b**) Ribbon representation of the top (**a**) and the side (**b**) views of the structure. One copy of Pnkp1, Rnl and Hen1 are coloured the same as in Fig. 1a, and the second copy of Pnkp1, Rnl and Hen1 are coloured sand, dark blue and ruby, respectively. (**c**,**d**) Surface of the Pnkp1–Rnl–Hen1 heterohexamer in the same colours and orientations as in **a** and **b**, respectively. Two arrows in **d** indicate the likely directions from which the damaged RNAs approach the Pnkp1–Rnl–Hen1 heterohexamer for RNA repair.

We crystallized the Pnkp1–Rnl–Hen1 heterohexamer and solved the structure at 3.3 Å resolution (Fig. 3; Supplementary Fig. 7; Table 1). The structure confirmed several observations from the chromatographic experiments, including the formation of Pnkp1 homodimer, Pnkp1–Rnl interaction (Fig. 1b, panel 1) and Rnl–Hen1 interaction (Fig. 1b, panel 3). Unexpectedly, Pnkp1 and Hen1 were found to make physical contact in the structure, which was not the case with the chromatographic analysis (Fig. 1b, panel 2). As a result of the additional Pnkp1–Hen1 interaction, each protein makes physical contacts with the other two proteins, resulting in the Pnkp1–Rnl–Hen1 heterotrimer adopting a ring structure. Because Pnkp1 forms a homodimer (Fig. 3, coloured cyan and sand), the overall architecture of the Pnkp1–Rnl–Hen1 heterohexamer consists of two rhomboid-shaped ring structures of Pnkp1–Rnl–Hen1 heterotrimers fused at the Pnkp1 dimer interface (Fig. 3a,c). The side view of the structure displays C2 symmetry (Fig. 3b,d), suggesting the presence of two functional units of the RNA repair machinery having RNA substrates approach from opposite directions (Fig. 3d, indicated by arrows).

On the local level, the Pnkp1 homodimer is formed by interactions between the same enzymatic domains, resulting in kinase and phosphatase dimer modules (Fig. 3a). With the exception of two short peptides (the N terminus and the link between the kinase and phosphatase domains), which span across the modules, the kinase and phosphatase modules essentially do not make contact with each other. Therefore, the relative orientation of the kinase and phosphatase modules could be flexible when Pnkp1 homodimer is alone. In the structure of the Pnkp1–Rnl–Hen1 heterohexamer, however, the relative orientation of the kinase and phosphatase modules is locked at 130° (Supplementary Fig. 8). This presumably results from Pnkp1 interacting with both Rnl and Hen1.

For the Pnkp1–Rnl interaction, each copy of Rnl makes contact with a single copy of Pnkp1 at the kinase domain. On the other hand, each copy of Hen1 make contacts with both copies of Pnkp1. Finally, despite little sequence similarities, the mode of interaction between Rnl and Hen1 in the Pnkp1–Rnl–Hen1 heterohexamer is similar to the one observed previously in the ligase module of the Pnkp–Hen1 RNA repair complex^24^, indicating that these two RNA repair complexes might be evolutionary related.

To assess the relative contribution of each protein–protein interaction to the stability of the overall complex, we calculated the surface area buried at each protein interface. The most extensive interaction was found at the interface of the Pnkp1 homodimer, with ∼6,800 Å^2^ total solvent-accessible surface area buried. This is followed by the Rnl–Hen1 interface (∼3,200 Å^2^ buried surface area), which is presumably responsible for the ligase activation in Rnl by Hen1 based on our previous study of the Pnkp–Hen1 RNA repair system^24^. Pnkp1 interacts with Rnl and Hen1 to approximately the same extent, with ∼600 and ∼700 Å^2^ buried surface areas, respectively.

### Molecular recognitions among Pnkp1, Rnl and Hen1

Formation of the Pnkp1–Rnl–Hen1 heterohexamer requires recognition between the kinase and phosphatase domains of Pnkp1, Pnkp1 and Rnl, Rnl and Hen1, and Pnkp1 and Hen1. The crystal structure of the Pnkp1–Rnl–Hen1 heterohexamer revealed the molecular basis for these recognitions as described below.

**Figure 4:**
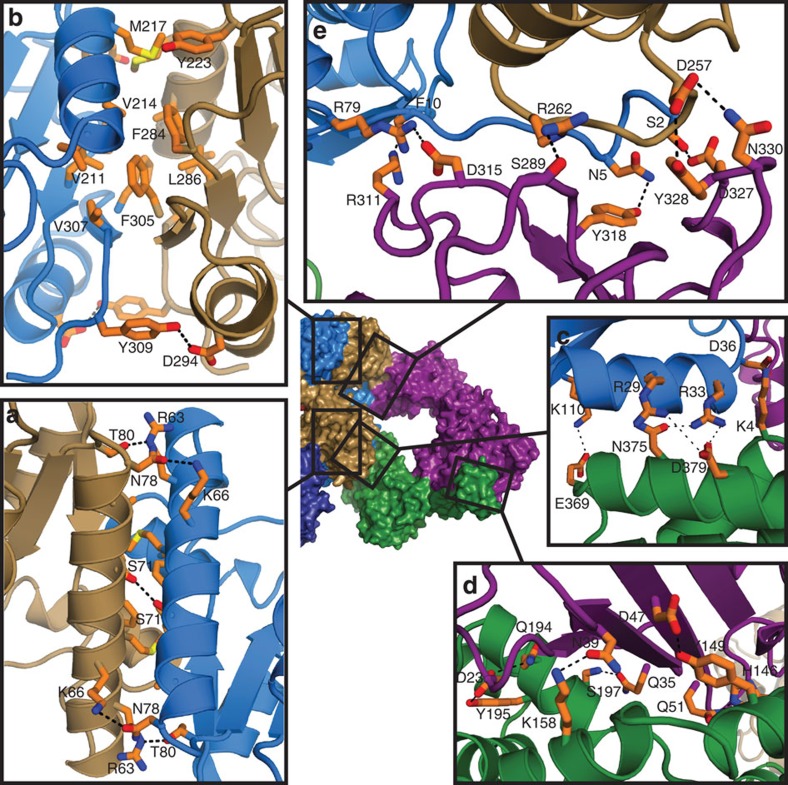
Details at the protein–protein interfaces. (**a**) Details of the interface between the two kinase domains of the Pnkp1 homodimer. Cα-chains of the structure are represented and coloured the same as in Fig. 3a,b. The side chains are in stick and coloured orange except heteroatoms, which are coloured individually (nitrogen in blue, oxygen in red and sulfur in yellow). Hydrogen bonds and salt bridges (3.5 Å or less) are depicted with black dashed lines. (**b**–**e**) Details of the interface between the two phosphatase domains of the Pnkp1 homodimer (**b**), Pnkp1 and Rnl (**c**), Rnl and Hen1 (**d**), and Pnkp1 and Hen1 (**e**).

The formation of the kinase module of Pnkp1 is via interactions between two kinase domains with antiparallel orientations (Fig. 4a). The interaction is mainly hydrophobic, with the side chains of V39, F46, M49, R63, M67 and L75 from both kinase domains forming the hydrophobic core at the dimer interface (Fig. 4a). The hydrophobic interaction is further enhanced by several hydrogen bonds, including one at the centre, and two at each end (Fig. 4a). As expected, DALI^26^ search revealed that the fold of the kinase domain of Pnkp1 is similar to the one seen in several structures of *T4*Pnkp^27^,^28^,^29^. In addition, the mode of dimerization was also observed in one of the *T4*Pnkp structures^29^ (Supplementary Fig. 9b), but the majority of the amino acids involved in dimerization are different (Supplementary Fig. 9a).

Unlike the kinase domains, formation of the phosphatase module results from interactions between two phosphatase domains oriented in parallel (Fig. 4b). Formation of a hydrophobic core, provided by the side chains of V211, V214, M217, Y223, F284, L286, F305 and V307, is also the main driving force for the dimerization (Fig. 4b). The interaction is further enhanced by two hydrogen bonds located at the bottom of the dimer interface. As in the case of kinase domain, DALI search revealed that the fold of the phosphatase domain is very similar to the one found in *T4*Pnkp^27^,^28^,^29^. Furthermore, the mode of dimerization of the phosphatase domains was also observed in one of the *T4*Pnkp structures^29^ (Supplementary Fig. 9c). Unlike the dimerization of the kinase domains, however, residues responsible for the dimer formation of the phosphatase domains are highly conserved (Supplementary Fig. 9a). These conservations, together with the study that mutations of residues at the dimer interface of *T4*Pnkp ablate the phosphatase activity^29^, indicate that the mode of dimerization of the phosphatase domains of Pnkp1 might be functionally important for phosphatase activity.

Each Rnl only makes contact with one kinase domain of the Pnkp1 homodimer, and the interaction is mainly electrostatic. The main interactions are the salt bridges formed between the positively charged residues in Pnkp1 (R29, R33 and K110) and the negatively charged residues in Rnl (E369 and D379) (Fig. 4c). An additional salt bridge of the opposite polarity is also formed between the side chains of D36 in Pnkp1 and K4 in Rnl. The side chain of R33 in Pnkp1 also forms a hydrogen bond with the side chain of N375 in Rnl (Fig. 4c).

As was previously observed in the structure of the ligase module of the Pnkp–Hen1 RNA repair complex^24^, recognition of Rnl by Hen1, which is presumably the molecular basis of ligase activation in Rnl, mainly occurs at two locations. First, part of the β-sheet of the N-terminal ligase-activating domain of Hen1 recognizes the insertion domain of Rnl via an extensive hydrogen-bonding network (Fig. 4d). Second, an extended loop of the N-terminal ligase-activating domain of Hen1 reaches over the dimer interface and interacts with the C-terminal part of Rnl via formation of several hydrogen bonds (Supplementary Fig. 10). Although the overall mode of Rnl–Hen1 interaction is similar to the one found in the ligase module of the Pnkp–Hen1 RNA repair complex^24^, substantial differences are present in terms of both overall structure and detailed interactions (Supplementary Fig. 11). The differences are particularly pronounced between the insertion domains of Pnkp and Rnl as well as between the N-terminal ligase-activating domains of Hen1, implying an early evolutionary divergence of these two RNA repair systems.

Recognition of Pnkp1 by Hen1, which was not observed in solution with the binary mixture of Pnkp1 and Hen1 (Fig. 1b, panel 2), is achieved with residues from Hen1 forming hydrogen bonds with residues from both copies of Pnkp1 (Fig. 4e). The residues responsible for interaction in Hen1 are mainly located in a 20-amino-acid peptide segment facing Pnkp1. The contacting residues from one copy of Pnkp1 belong to the kinase domain (Fig. 4e, coloured cyan), and those contributed by the other copy of Pnkp1 are from the phosphatase domain (Fig. 4e, coloured sand).

### Implications for the mechanism of RNA repair

To understand why the RNA repair complex requires two copies of each protein, the two units of the Pnkp1–Rnl–Hen1 heterotrimer must first be defined based on the expected sequential events of protein synthesis and heterohexamer assembly *in vivo*. As shown in Supplementary Fig. 3b, Rnl and Pnkp1 are co-translated in *C. gingivalis* (with Rnl being synthesized first), whereas Hen1 is synthesized separately. Therefore, Rnl and Pnkp1 should form a complex first, followed by association with Hen1. Thus, the copy of Rnl and Pnkp1 that make physical contact, together with the copy of Hen1 that interacts with the Rnl, should be assigned to the same unit of the Pnkp1–Rnl–Hen1 heterotrimer. Labelling of the six proteins of the Pnkp1–Rnl–Hen1 heterohexamer shown in Fig. 3a takes into consideration these *in vivo* sequential events.

**Figure 5:**
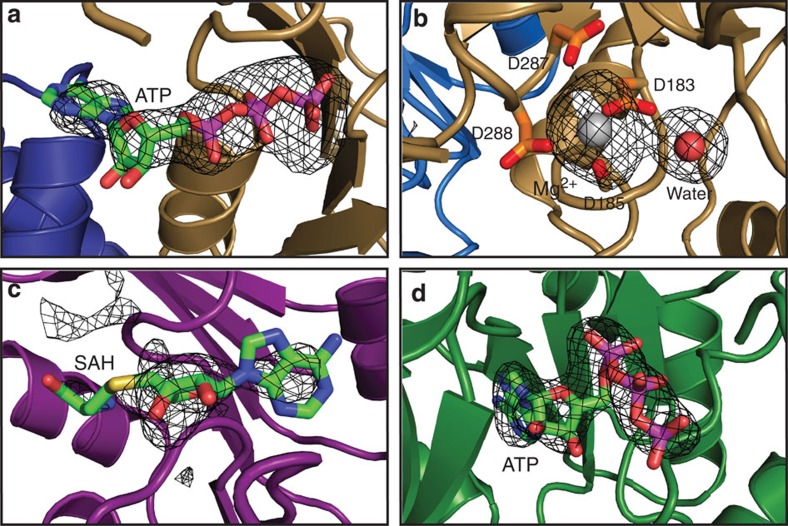
The presence of cofactors in all enzymatic active sites. (**a**) ATP bound in the kinase active site of Pnkp1-a. Proteins are depicted and coloured the same as in Fig. 3a,b. ATP is in stick and coloured green with the exception of heteroatoms, which are coloured individually (nitrogen in blue, oxygen in red, phosphate in magenta and sulfur in yellow). The simulated annealing composite *2mFo-DFc* omit density map contoured at 1.5σ is in black mesh. (**b**) A magnesium ion bound in the phosphatase active site of Pnkp1-a. Mg^2+^ depicted in sphere and coloured silver. A water molecule (in red sphere) was tentatively modelled on additional electron density 4.5 Å away from Mg^2+^, which could also be a phosphate group with a reduced occupancy. (**c**) SAH was modelled in the methyltransferase active site of Hen1-b. Because SAM or SAH was not added during crystallization or crystal soaking, the SAH modelled in the methyltransferase active site was likely obtained by Hen1 during its overexpression in *E. coli.* A significantly weaker omit map compared with other cofactors indicates that only a small fraction of SAH is retained by Hen1 after protein purification and crystallization steps. (**d**) ATP bound in the ligase active site of Rnl-b.

**Figure 6:**
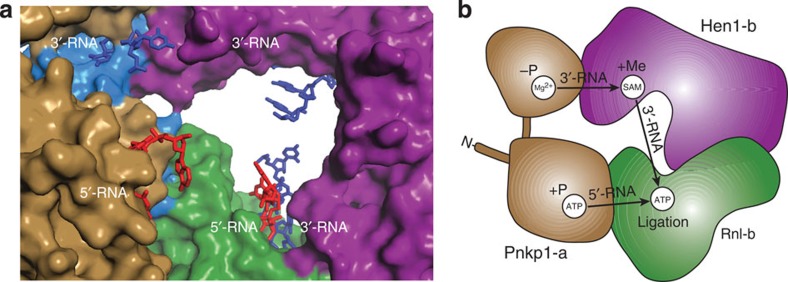
Docking model and proposed mechanism of RNA repair carried out by the Pnkp1–Rnl–Hen1 heterohexamer. (**a**) Modelling the 5′ end (red) and the 3′-end (blue) of damaged RNAs into all four active sites required for RNA repair. The modelled RNAs are in stick, and the proteins are depicted the same as in Fig. 3c but with a closer view on the vacant space surrounded by the b-unit of the Pnkp1–Rnl–Hen1 heterotrimer (coloured cyan, green and magenta) and Pnkp1-a (coloured sand). (**b**) Schematic view of Pnkp1, Rnl and Hen1 that provide four active sites for RNA repair. Pnkp1-b is omitted for clarity. The locations of the active sites are indicated by cofactors (white circles), with ATP in the kinase and ligase active sites, a magnesium ion in the phosphatase site and SAM in the methyltransferase site. Arrows indicate the travel pathways for both the 5′ end and 3′ end of a damaged RNA for RNA repair.

To provide insight into the mechanism of RNA repair by the Pnkp1–Rnl–Hen1 heterohexamer, we soaked crystals with adenosine triphosphate (ATP) before data collection, which resulted in a crystal structure with cofactors occupying all active sites (Fig. 5). Guided by the locations of these cofactors, and aided by the published structural homologues of Pnkp1 and Hen1 in complex with nucleic acids^28^,^30^,^31^ as well as the model of RNA in complex with the ligase module of Pnkp–Hen1^24^, we manually docked short single-stranded RNAs into the four active sites located in the inner rim of one ring structure (Fig. 6a; Supplementary Fig. 12). The docking model shows that, while the reacting ends of single-stranded RNAs (not observed in Fig. 6a) are placed in the active site, the opposite ends point to a vacant space at the centre of the ring structure, indicating that a RNA substrate approaches each repair unit from this vacant space for RNA repair. Therefore, we propose a mechanism for RNA repair carried out by the Pnkp1–Rnl–Hen1 heterohexamer as schematically depicted in Fig. 6b.

The damaged RNA might approach the four active sites for repair from the side of each ring, as indicated by arrows shown in Fig. 3d. The 5′ end of the damaged RNA would first enter the kinase site for phosphorylation and, at the same time, the 3′-end of the damaged RNA would go into the phosphatase site for dephosphorylation. Because of highly efficient 5′-phosphorylation carried out by the kinase domain of Pnkp1 (Fig. 2a), the 5′ end would be the first to enter the ligase site, where it could be activated with 5′-adenylation by RNA ligase. On the other hand, after 3′-dephosphorylation carried out by the phosphatase domain of Pnkp1, the 3′ end could make a detour to the methyltransferase site of Hen1 for 2′-*O*-methylation before joining the 5′ end in the ligase site. With the presence of both processed ends in the ligase site, RNA ligation occurs, which results in repaired RNA with 2′-*O*-methylation.

Because RNA repair requires four different enzymatic activities, we suggest a possibility of a single RNA repair event performed by the four active sites located in the same inner rim. Utilizing four active sites contributed from both rings for a repair event would demand that an RNA substrate accesses active sites far away from each other, which would likely require the RNA substrate to associate with and dissociate from the RNA repair complex more than once. As described previously, efficient 2′-*O*-methylation of the repaired RNA is desired due to its immunity to future damage. RNA repair performed by the four active sites shown in Fig. 6b would allow this to be achieved.

If our mechanistic hypothesis described above stands, the structure of the Pnkp1–Rnl–Hen1 heterohexamer provides an explanation for the requirement for two copies of each active site in the RNA repair complex. Among the four active sites located on the inner rim of the same ring structure, the kinase and phosphatase active sites are from one unit of the Pnkp1–Rnl–Hen1 heterotrimer, and the methyltransferase and ligase active sites are contributed from the other unit (Figs 5 and 6). A single Pnkp1–Rnl–Hen1 heterotrimer, although possessing all four different active sites required for RNA repair, would have placed active sites far away from each other with opposite orientations.

The relative locations of the four active sites and the size of the vacant space surrounded by the four active sites could be responsible for efficient 2′-*O*-methylation during RNA repair. Our structure revealed that the methyltransferase site is located between the phosphatase and ligase sites, providing the 3′ end of the damaged RNA maximal opportunity to be 2′-*O*-methylated after dephosphorylation but before ligation. It is possible that the 3′ end of the damaged RNA might skip the methyltransferase site and be transferred directly from the phosphatase to ligase sites. Such an event might occur occasionally, as we have observed that 2′-*O*-methylation is not complete for all repaired RNA^22^. However, the space available to manoeuvre both ends of the damaged RNA is in fact small. Therefore, if the 5′ end occupies the active site of the ligase as supported by the kinetic analyses, the most likely pathway for the 3′ end of the damaged RNA would be phosphatase→methyltransferase→ligase instead of phosphatase→ligase due to the spatial constraint for the movement of RNA. In addition, we cannot rule out the possibility that the topology and the surface charge of the phosphatase→methyltransferase→ligase route have evolved to facilitate the entrance of the 3′ end into the methyltransferase active site after dephosphorylation but before ligation. Additional studies are required to investigate such a possibility.

## Discussion

In this study, we described the discovery of a new RNA repair system in bacteria, which was subsequently biochemically and structurally characterized. In addition to providing mechanistic insight into RNA repair by the Pnkp1–Rnl–Hen1 complex, the study may have broader implications discussed below.

The study of the Pnkp1–Rnl–Hen1 RNA repair complex may provide insight into how RNA is repaired by the bacterial Pnkp–Hen1 RNA repair complex, whose structure is unknown. As illustrated in Supplementary Figs 2 and 11, these two RNA repair complexes are clearly evolutionary related. Therefore, we expect the Pnkp–Hen1 heterotetramer to be similar in structure to the portion of the C-terminal half of Pnkp in complex with Hen1 (Pnkp-C–Hen1). On the other hand, the manner in which the two units of Pnkp-C–Hen1 are brought together by Pnkp-N to form the Pnkp–Hen1 heterotetramer is less clear. Unlike Rnl of the Pnkp1–Rnl–Hen1 complex, which makes physical contact with the kinase domain of Pnkp1, Pnkp-C directly connects to the phosphatase domain of Pnkp-N (Supplementary Fig. 2b). Furthermore, the phosphatase domains of these two RNA repair complexes belong to two completely different superfamilies (Supplementary Fig. 2). Therefore, the structures corresponding to the kinase and phosphatase domains of these two RNA repair complexes are expected to be different. Nevertheless, the fact that Pnkp and Hen1 form a heterotetramer—thus possessing the same number of active sites as the Pnkp1–Rnl–Hen1 heterohexamer—indicates the likely formation of two ring structures. Therefore, the mechanism of RNA repair suggested by the structure of the Pnkp1–Rnl–Hen1 heterohexamer is likely to be conserved in the Pnkp–Hen1 heterotetramer.

The study described here may also shed some light on the likely *in vivo* RNA substrates and the nature of RNA repair carried out by the complex. Despite extensive *in vitro* studies of the Pnkp–Hen1 as well as the new Pnkp1–Rnl–Hen1 RNA repair complexes, the *in vivo* RNA substrates still remain unknown. The difficulties of revealing *in vivo* RNA substrates stem from the assumption that RNA damage and repair most likely occur when organisms are in the wilderness, where they are exposed to ribotoxins released by nearby species. Therefore, the events of RNA damage and repair occurring in the wilderness are difficult to reproduce in a laboratory setting. The crystal structure of the Pnkp1–Rnl–Hen1 heterohexamer described here indicates that the four active sites required for repair appear to be readily accessible to a variety of damaged RNAs. This is consistent with our previous *in vitro* biochemical studies that the Pnkp–Hen1 RNA repair complex exhibits broad substrate specificity^25^. Therefore, instead of repairing a particular RNA damaged by a particular ribotoxin, the Pnkp–Hen1 and Pnkp1–Rnl–Hen1 RNA repair complexes might be generic RNA repair systems that repair a variety of damaged RNAs *in vivo*.

**Figure 7:**
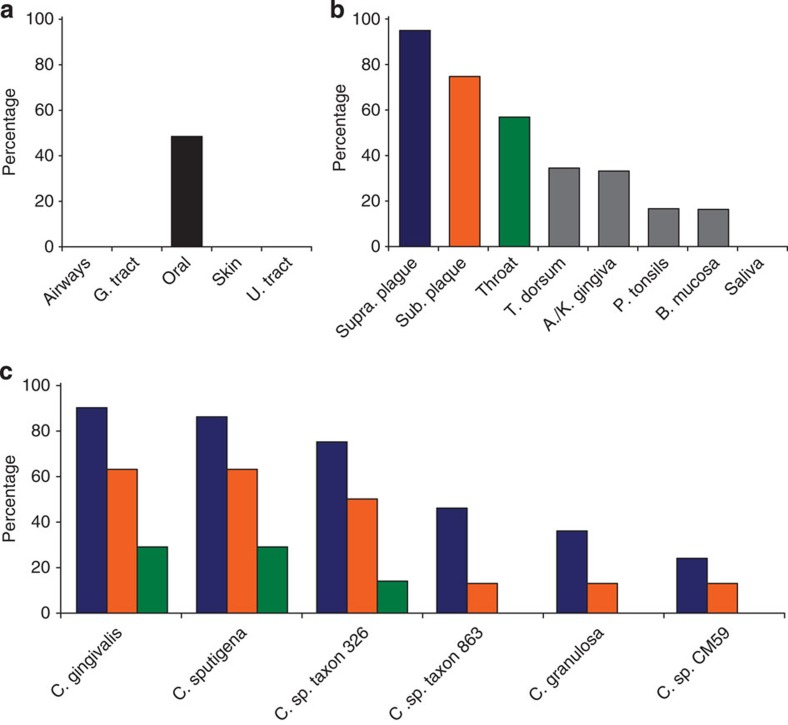
Presence of the Pnkp1–Rnl–Hen1 RNA repair complex in microbes living in humans. (**a**) Distribution of bacteria possessing the Pnkp1–Rnl–Hen1 RNA repair complex at five different locations of the human body. G. tract, gastrointestinal tract; U. tract, urogenital tract. (**b**) Distribution of the bacteria at different sub-locations of the human mouth. Supra. plaque, supragingival plaque; Sub. plaque, subgingival plaque; T. dorsum, tongue dorsum; A/K gingival, attached/keratinized gingival; P. tonsils, palatine tonsils; B. mucosa, buccal mocosa. (**c**) Distribution of six human-hosted bacterial species possessing the Pnkp1–Rnl–Hen1 RNA repair complex at three major sub-locations of the human mouth.

Despite having little knowledge about the *in vivo* biological functions of RNA repair carried out by the Pnkp1–Rnl–Hen1 heterohexamer, we were able to identify 10 bacterial species that possess the Pnkp1–Rnl–Hen1 RNA repair complex. Furthermore, based on the analyses of the data released by the Human Microbiome Project (HMP), we were able to locate an ecosystem for many of them. Among 10 bacterial species, six were isolated from human, one from the mouth of a dog and three were from environmental samples with unknown sources. Metagenomic analyses using the data released by HMP revealed that, of five locations in humans where samples were collected, bacteria possessing the RNA repair complex were only found in the mouth (Fig. 7a). More detailed analysis of the samples collected from different locations of the human mouth identified that these bacteria mainly live in gingival plaques (Fig. 7b, blue and orange bars). Further analysis of the data based on the search for individual bacterium indicated that *C. gingivalis*, *C. sputigena* and *Capnocytophaga sp. taxon 326* are the most abundant (Fig. 7c). It is unclear why among ∼5,000 bacterial species with known genomes, only a subset of *Capnocytophaga* species that mainly live in gingival plaques of the human mouth possess a Pnkp1–Rnl–Hen1 RNA repair complex. Both *C. gingivalis* and *C. sputigena* have been implicated in periodontal and dental diseases^32^,^33^,^34^. Therefore, if the unique RNA repair carried out by the Pnkp1–Rnl–Hen1 complex in these bacteria provides them with a heightened ability to survive, inhibiting RNA repair in these bacteria may provide a vehicle to reduce the population of certain pathogens.

## Methods

### Expression and purification of *Cgi*Pnkp1, *Cgi*Rnl and *Cgi*Hen1

DNA corresponding to Pnkp1, Rnl and Hen1 were amplified from the *C. gingivalis* genomic DNA purchased from ATCC. The DNA sequences of the primers used for PCR amplifications are listed in Supplementary Table 1. The amplified PCR products were digested by restriction enzymes, and the digested products were inserted into the pETDuet-1 vector via DNA ligation. The encoding plasmids were transformed into *Escherichia coli* BL-21(DE3) strain individually, and the proteins were expressed at 18 °C for 20 h after induction with 0.5 mM isopropyl-b-D-thiogalactoside (IPTG). Cells were harvested by centrifugation and stored at −80 °C. Thawed cell pellets were resuspended in lysis buffer (20 mM Tris-HCl, pH 8.0, 10 mM NaCl, 2% glycerol and 1 mM dithiothreitol (DTT)) and lysed using the French press. The cell lysate was centrifuged and the proteins were purified from the supernatant by a fast protein liquid chromatography (FPLC) system. Pnkp1 was purified to homogeneity using diethylaminoethanol (DEAE) ion exchange, heparin affinity, Mono Q ion exchange and Superdex 200 size-exclusion chromatography. Rnl was purified the same as Pnkp1, except the Mono Q ion exchange step was omitted. Hen1 was purified similarly as Pnkp1, except heparin affinity chromatography was omitted. All purified proteins were stored in the gel filtration buffer (10 mM HEPES, pH 7.0, 200 mM NaCl and 1 mM DTT) for further study.

To produce selenomethione-incorporated Pnkp1, the *E. coli* Rosetta strain was used for expression and methionine pathway inhibition was used for cell growth. The protein was purified the same as the wild-type Pnkp1 described above.

### *In vitro* reconstitution and kinetic studies

The purified recombinant proteins were analysed individually using size-exclusion chromatography. To assess protein–protein interaction, two different proteins were combined in equimolar and incubated at 4 °C for an hour before size-exclusion chromatography. To assemble the entire RNA repair complex, all three proteins were mixed in equimolar concentrations, incubated at 4 °C for an hour and analysed by size-exclusion chromatography.

To carry out RNA repair, ^33^P-internally radiolabelled and colicin D-cleaved tRNA^Arg^ was prepared as described previously^22^. In a 20-μl reaction volume, the cleaved tRNA (4 μM) was incubated with different combinations of proteins (1 μM each) as shown in Fig. 1c in Repair buffer (25 mM Tris-HCl, pH 8.0, 50 mM KCl, 2.5 mM MgCl_2_, 0.5 mM MnCl_2_, 0.05 mM EDTA, 5 mM DTT and 2.5% glycerol) in the presence of 0.2 mM ATP and 0.05 mM AdoMet at 37 °C for 45 min. After the reaction, the sample was processed with phenol extraction followed by ethanol precipitation to recover RNA. RNA was dissolved in DPAGE loading buffer, heated at 95 °C for 3 min to denature RNA and the sample was analysed by 15% DPAGE. The radioactivity of the repaired and unrepaired tRNAs was visualized using a PhosphorImager system (Molecular Dynamics).

For kinetic studies, tRNA^Arg^-ΔT was employed, which allowed 5′-half (38 nt) and 3′-half (22 nt) RNAs to be purified after cleavage by colicin D. The 5′-phosphoylation reaction was carried out in a 30-μl reaction volume containing 10 μM 3′-half RNA, 0.025 μM enzyme (*Cgi*Pnkp1 homodimer or *Cgi*Pnkp1–Rnl–Hen1 heterohexamer) and 0.2 mM ^33^P-α-ATP in Repair buffer at 37 °C. Five μl of reaction solution was taken out at the time points of 0, 5, 15, 30 and 60 min, and the samples were processed and analysed analogous to the ones in the repair assay described above.

To carry out the 3′-dephosphorylation reaction, the 5′-half RNA was prepared with RNA repair by *Cgi*Pnkp1–Rnl–Hen1 heterohexamer in the presence of 0.2 mM ^33^P-γ-ATP (to introduce the radiolabelled phosphate at the junction of the repaired RNA), followed by re-cleavage with colicin D. The 3′-dephosphoylation reaction was carried out analogous to one of the kinase reactions, except that the RNA substrate was 1.0 μM, 5′-half RNA was radiolabelled at the 2′,3′-cyclic phosphate, the concentration of the enzyme was 0.25 μM and no ATP was added.

The published protocol employed for the studies of the Pnkp–Hen1 RNA repair complex was used for RNA ligation reaction without and with SAM, except that the processed RNA substrates were used in this study^22^. The same protocol was used for re-cleavage assays of the repaired RNA by colicin D.

### Crystallization, data collection and structural determination

The purified Pnkp1–Rnl–Hen1 heterohexamer was concentrated to 7 mg ml^−1^ and mixed with a reservoir solution containing 8% PEG 6000, 0.2 M NaCl, 15 mM MgCl_2_ and 100 mM MES (pH 6.2). Full-size crystals of the Pnkp1–Rnl–Hen1 heterohexamer grew using the hanging drop vapour diffusion method at 4 °C in 2 weeks. Crystals were soaked in a series of cryoprotecting solutions containing all the components of the reservoir solution supplemented with increasing percentage of glycerol to a final concentration of 30%. For the data set that produced the structure of the Pnkp1–Rnl–Hen1 heterohexamer in complex with ATP, 5 mM ATP was also included in crystal soaking. The cryoprotected crystals were mounted in nylon loops and flash-frozen in liquid nitrogen. Data were collected at 21-ID beamline at the Advanced Photon Source and processed by HKL2000 (ref. 35).

Phase for the structure of the hexamer was determined based on single-wavelength anomalous diffraction (SAD) data from a crystal of the heterohexamer with the selenium-containing Pnkp1. Of 18 selenomethionine residues present in Pnkp1 homodimer, 17 of them were found with AutoSol of the Phenix software^36^. The figure of merit was 0.23 before the density modification, and a portion of the density-modified SAD electron density map was shown in Supplementary Fig. 7. A significant portion of the model was automatically built by the Phenix program, and the remaining model was manually built using Coot program^37^. Many rounds of model building, followed by refinement using the Phenix program, resulted in a final model of the Pnkp1–Rnl–Hen1 heterohexamer with *R*_work_ and *R*_free_ of 17.6% and 23.3%, respectively (Table 1).

### Metagenomic analyses

Amino-acid sequences of *Cgi*Hen1 of the Pnkp1–Rnl–Hen1 RNA repair complex were employed for a BLASTP search against metagenomic data released by the HMP (https://img.jgi.doe.gov/cgi-bin/imgm_hmp/main.cgi). The *E*-value for the search was 1e−50. Because of significant difference between Hen1 from the Pnkp1–Rnl–Hen1 RNA repair complex and the one from the Pnkp–Hen1 system, the *E*-value of 1e−50 ensures that a positive result from a search is an indication of the presence of the Pnkp1–Rnl–Hen1 RNA repair system, not the Pnkp–Hen1 system, in the data set used for the search.

The entire metagenomic analyses consist of three stages. First, BLASTP search using *Cgi*Hen1 was carried out against data sets of each of the five groups (airway, gastrointestinal tract, oral, skin and urogenital tract). Second, the search was carried out against data sets of each of the sub-groups of the human mouth. Third, BLASTP search using the sequences of Hen1 from six human-hosted bacteria was carried out one at a time against the data sets of supragingival plaque, subgingival plaque and throat. For the third search, the positive result of the search also required the sequence to be at least 97% identical to distinguish different Hen1 within these six bacterial species.

## Author contributions

P.W. carried out cloning, overexpression and purification of proteins with the help from K.S. P.W. performed *in vitro* reconstitution experiments. P.W. carried out crystallization. P.W., K.S. and R.H.H. carried out data collection. P.W. and R.H.H. carried out structural determination and model refinement. All authors contributed to the writing of the manuscript.

## Additional information

**Accession codes.** Coordinates and structure factors have been deposited in the Protein Data Bank with accession code 4XRP for the Pnkp1–Rnl–Hen1 heterohexamer alone, and 4XRU for the Pnkp1–Rnl–Hen1 heterohexamer in complex with cofactors.

**How to cite this article:** Wang, P. *et al.* Reconstitution and structure of a bacterial Pnkp1–Rnl–Hen1 RNA repair complex. *Nat. Commun.* 6:6876 doi: 10.1038/ncomms7876 (2015).

## Supplementary Material

Supplementary Information Supplementary Figures 1-12, Supplementary Table 1 and Supplementary References

## Figures and Tables

**Table 1:** Data collection and refinement statistics.

	**Pnkp1-Rnl-Hen1** **hexamer+cofactors**	**Pnkp1-Rnl-Hen1 hexamer**
*Data collection*		
Data type	SAD	Native
Space group	*P*2_1_	*P*2_1_
Cell dimensions		
*a*, *b*, *c* (Å)	108.5, 187.2, 112.0	111.2, 179.2, 114.3
*α*, *β*, *γ* (°)	90.0, 106.0, 90.0	90.0, 103.7, 90.0
Resolution (Å)	50.0–3.4	50.0–3.3
*R*_merge_ (%)	4.4	7.0
*I/σI*	17.1 (3.1)	13.9 (3.8)
Completeness (%)	98.4 (91.1)	99.8 (98.7)
Redundancy	7.5	5.1
		
*Refinement*		
Resolution (Å)	42.9–3.4	47.2–3.3
No. of reflections	57,526 (5,292)	65,456 (6,432)
*R*_work_/*R*_free_ (%)	17.5/23.8 (24.8/31.4)	17.6/23.3 (25.1/30.7)
No. of atoms	18,713	18,886
Protein	18,442	18,711
Ligand/ion	179	84
Water	92	91
B factors (Å^2^)		
Protein	85.4	71.3
Ligand/ion	109.1	97.6
Water	54.9	48.7
R.m.s. deviations		
Bond lengths (Å)	0.010	0.012
Bond angles (°)	1.55	1.64

Values in parenthesis are for the highest resolution shell.
