# Supplementary material for: Reconstitution and structure of a bacterial Pnkp1–Rnl–Hen1 RNA repair complex
Source: Nat Commun. 2015 Apr 17;6:6876. doi: 10.1038/ncomms7876 (PMC4411300; doi:10.1038/ncomms7876)
Supplement: Supplementary Information — Supplementary Figures 1-12, Supplementary Table 1 and Supplementary References [file ncomms7876-s1.pdf]

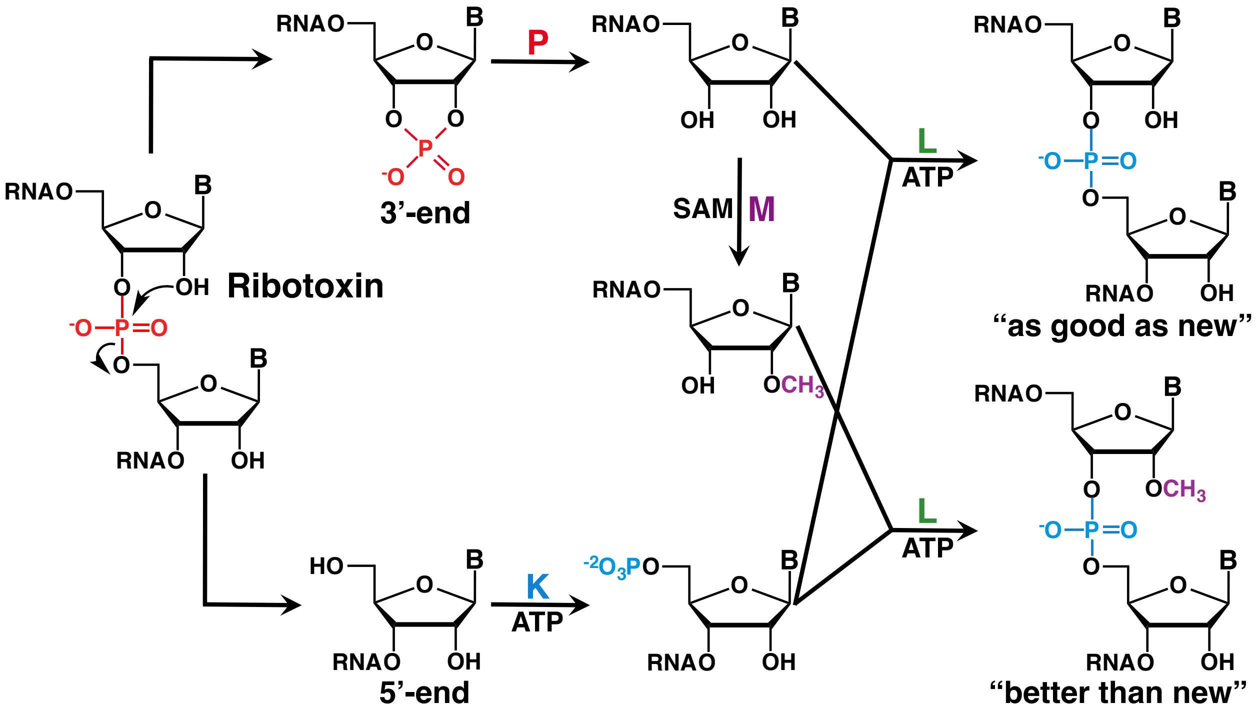

Supplementary Figure 1 | General mechanism of RNA damage and repair. In most cases, a ribotoxin activates the 2'-OH group of the targeted nucleotide, allowing it to carry out nucleophilic attack on the adjacent phosphate backbone to cleave RNA. The 5'-OH and the 2',3'-cyclic phosphate groups generated are not compatible with a classical RNA ligase. Therefore, both ends are processed by a kinase (**K**) and a phosphatase (**P**). After phosphorylation at the 5'-end and dephosphorylation at the 3'-end, the two processed ends are joined by a ligase (**L**) to restore the damaged RNA to its original form (as good as new). With the Pnkp–Hen1 and current Pnkp1–Rnl–Hen1 RNA repair complexes, however, the 2'-OH group responsible for the original RNA cleavage is methylated by a unique 3'-terminal 2'-O-methyltransferase (**M**) after dephosphorylation but before ligation, resulting in the repaired RNA resisting future cleavage by the same ribotoxin at the repair site (better than new).

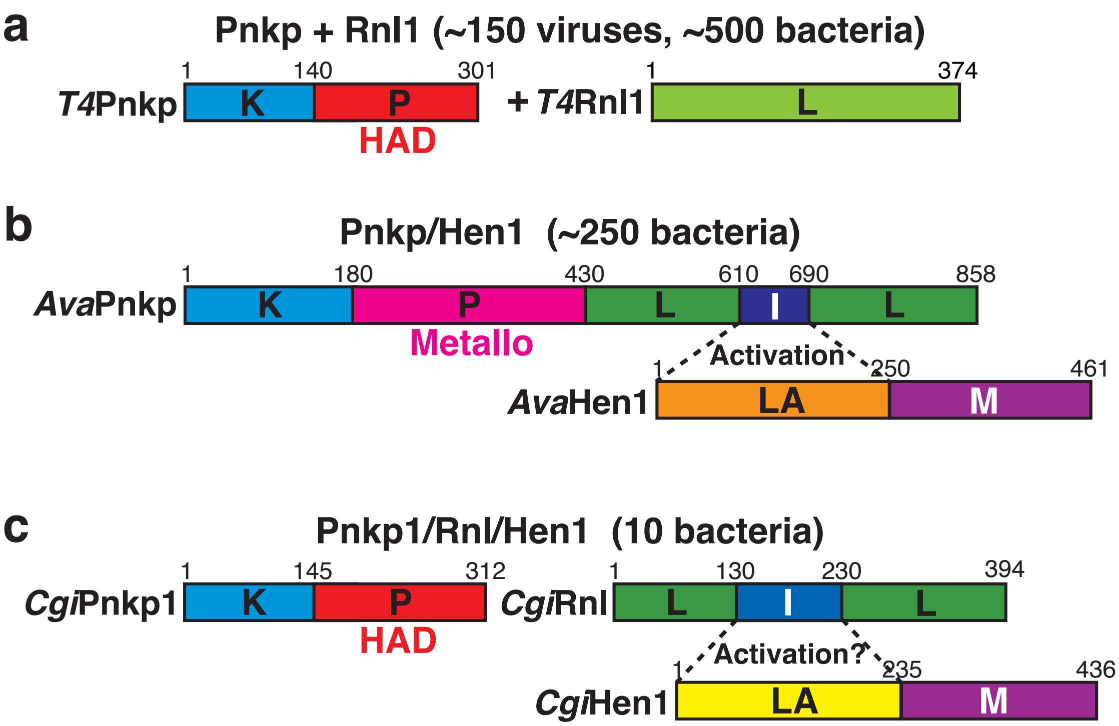

Supplementary Figure 2 | Overview of the newly discovered RNA repair system and its comparison to well-studied RNA repair systems. (**a**) Schematic view of the two proteins from bacteriophage T4 in RNA repair. Our bioinformatic analysis indicates that a similar RNA repair system is present in approximately 150 other viruses and 500 bacterial species, which requires experimental verification. (**b**) Schematic view of the Pnkp–Hen1 RNA repair complex from *Anabaena variabilis* (*Ava*), which was employed in our previous *in vitro* reconstitution[1]. The Pnkp–Hen1 RNA repair system can be found in about 250 bacterial species of different phyla. (**c**) The same view of the newly discovered Pnkp1–Rnl–Hen1 RNA repair complex from *Capnocytophaga gingivalis* (*Cgi*), presented in this study. This system is currently only found in ten bacteria belonging to Flavobacteriaceae family of Bacteroidetes phylum. Most domains of proteins are labeled and colored the same as in Supplementary Fig. 1 with the exception of two new additions: I, insertion domain; LA, ligase-activating domain. Different shades of green are employed for T4 and bacterial ligases to denote that they belong to different families of RNA ligases. Because the insertion domains (I) and the ligase-activating domains of the two bacterial RNA repair systems do not show apparent sequence homology, they are also depicted with different shades of blue (I) and colors (LA).

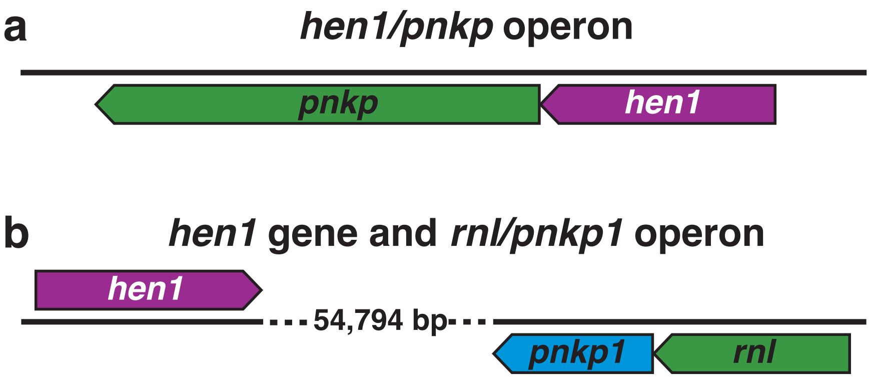

Supplementary Figure 3 | Gene organization of the two representative bacterial RNA repair systems depicted in Supplementary Fig. 2b,c. (**a**) The genes encoding two proteins of the *Ava*Pnkp–Hen1 RNA repair complex are present in the same operon. Both proteins are co-translational, with Hen1 being synthesized first. (**b**) The newly discovered *Cgi*Pnkp1–Rnl–Hen1 RNA repair complex has the gene encoding Hen1 separated from the ones encoding Rnl and Pnkp1. As in the case of *Ava*Hen1 and *Ava*Pnkp, *Cgi*Rnl and *Cgi*Pnkp1 are co-translational, with Rnl being synthesized first. 54,794 base-pairs (bp) of DNA separate the gene encoding Hen1 and the ones encoding Rnl and Pnkp1.

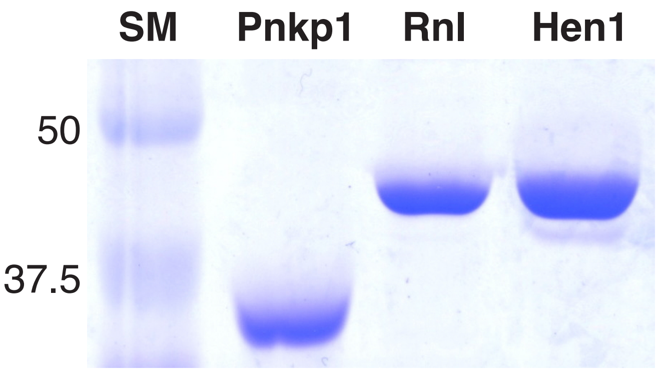

Supplementary Figure 4 | SDS gel analysis of the purified recombinant *Cgi*Pnkp1, *Cgi*Rnl, and *Cgi*Hen1. SM, size marker.

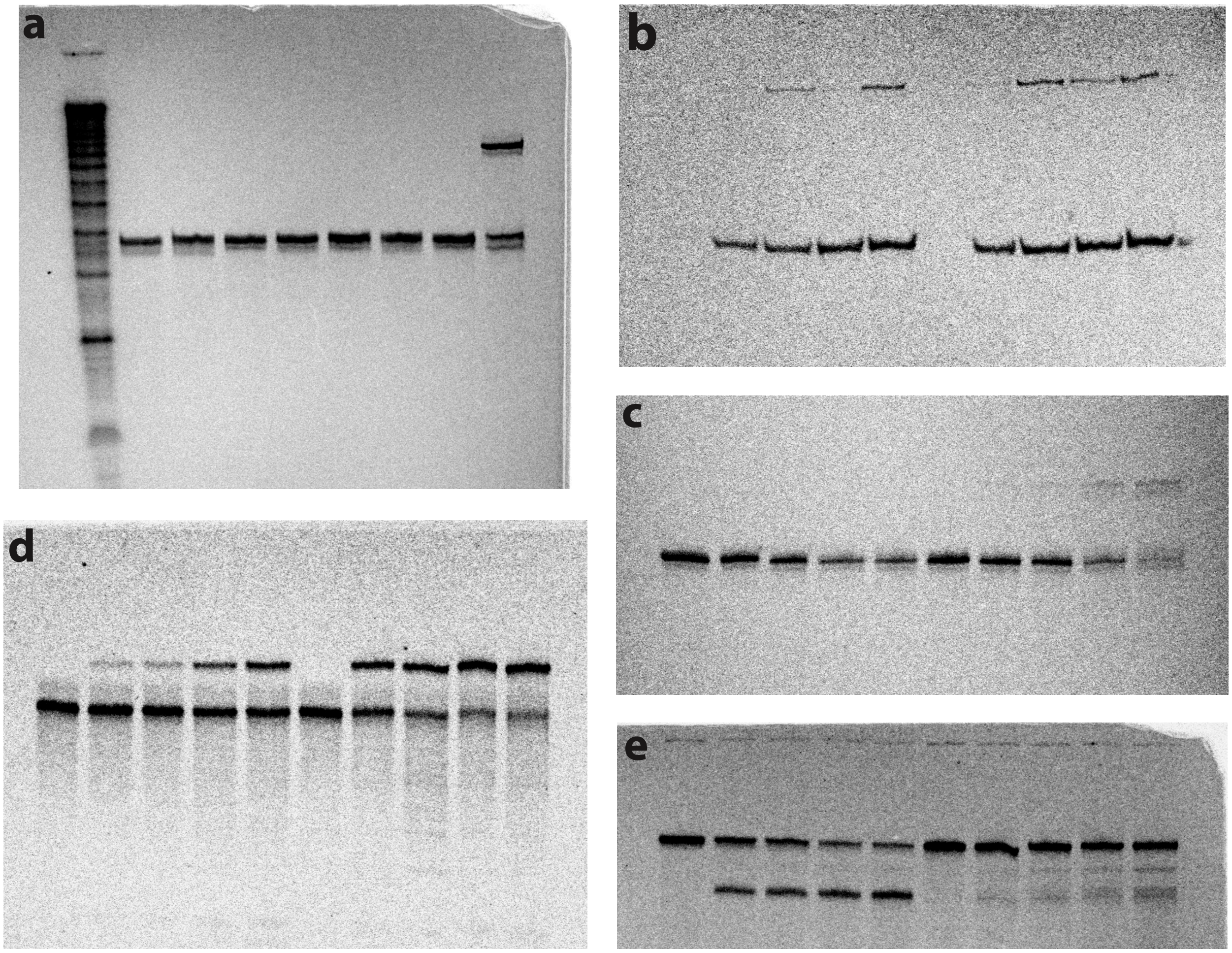

Supplementary Figure 5 | Broader views of DPAGE analyses of individual enzymatic reactions. The cropped versions of these images were used in Fig. 1c (**a**), Fig. 2a (**b**), Fig. 2b (**c**), Fig. 2c (**d**), and Fig. 2d (**e**).

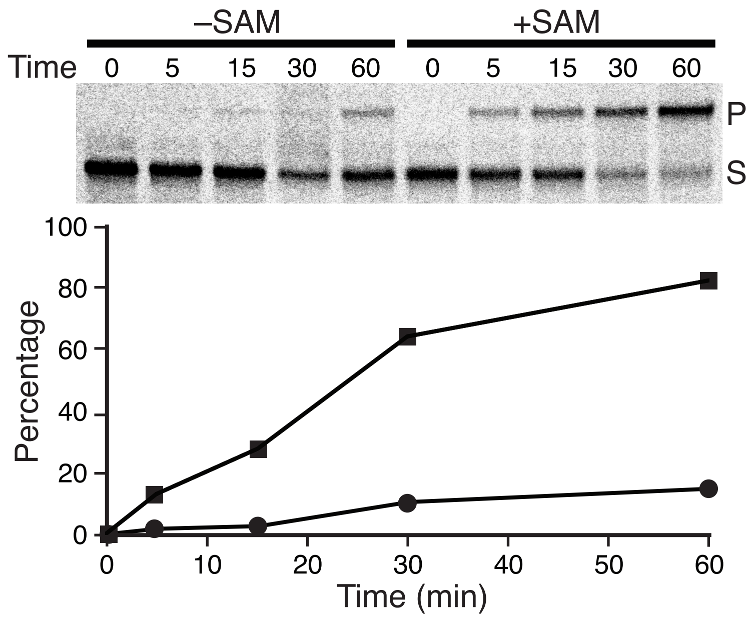

Supplementary Figure 6 | Kinetics of RNA ligation carried out by the Pnkp1–Rnl–Hen1 heterotetramer. The experiment was identical to the one shown in Fig. 2c with the exception that the Pnkp1–Rnl–Hen1heterotetramer replaces the Rnl–Hen1 heterodimer.

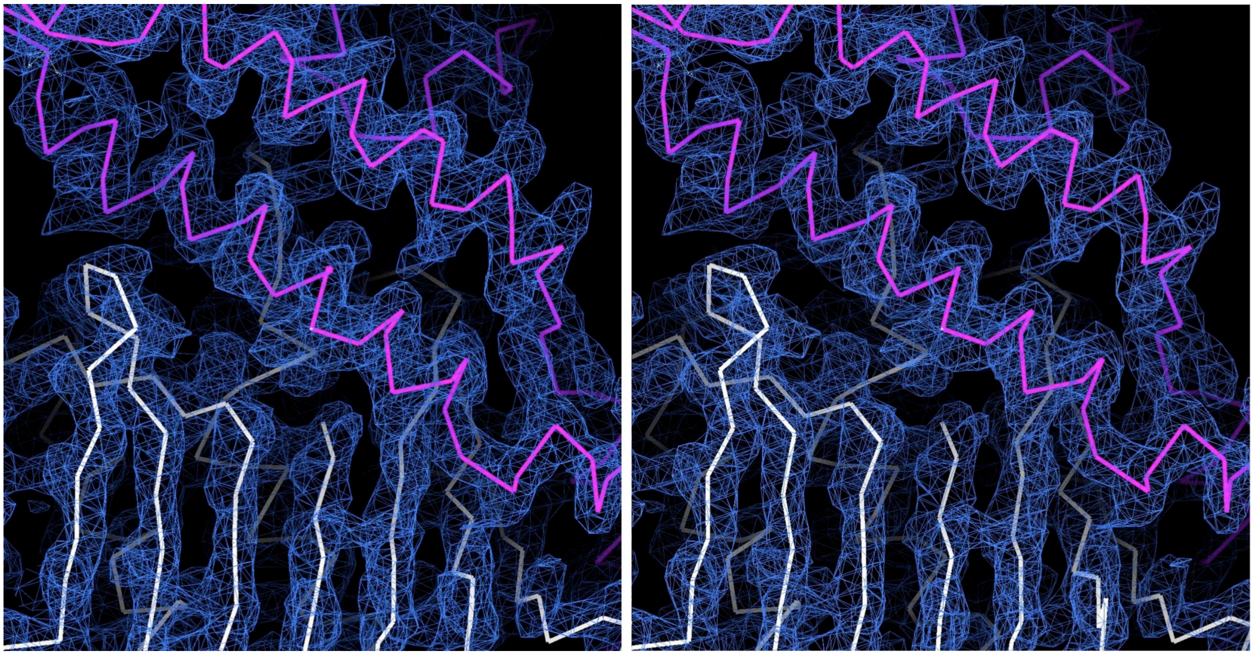

Supplementary Figure 7 | Stereoview of a portion of the electron density map after density modification. The map was centered at the Rnl–Hen1 dimer interface, with Rnl in magenta and Hen1 in white.

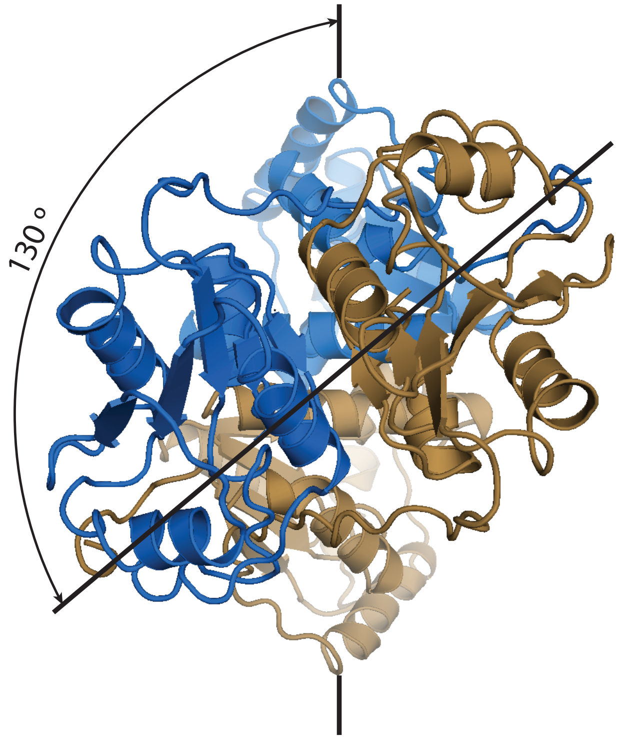

Supplementary Figure 8 | Relative orientations of the kinase and phosphatase modules of the Pnkp1 homodimer observed in the structure of the Pnkp1–Rnl–Hen1 heterohexamer. The structure is depicted and colored the same as in Fig. 3a,b.

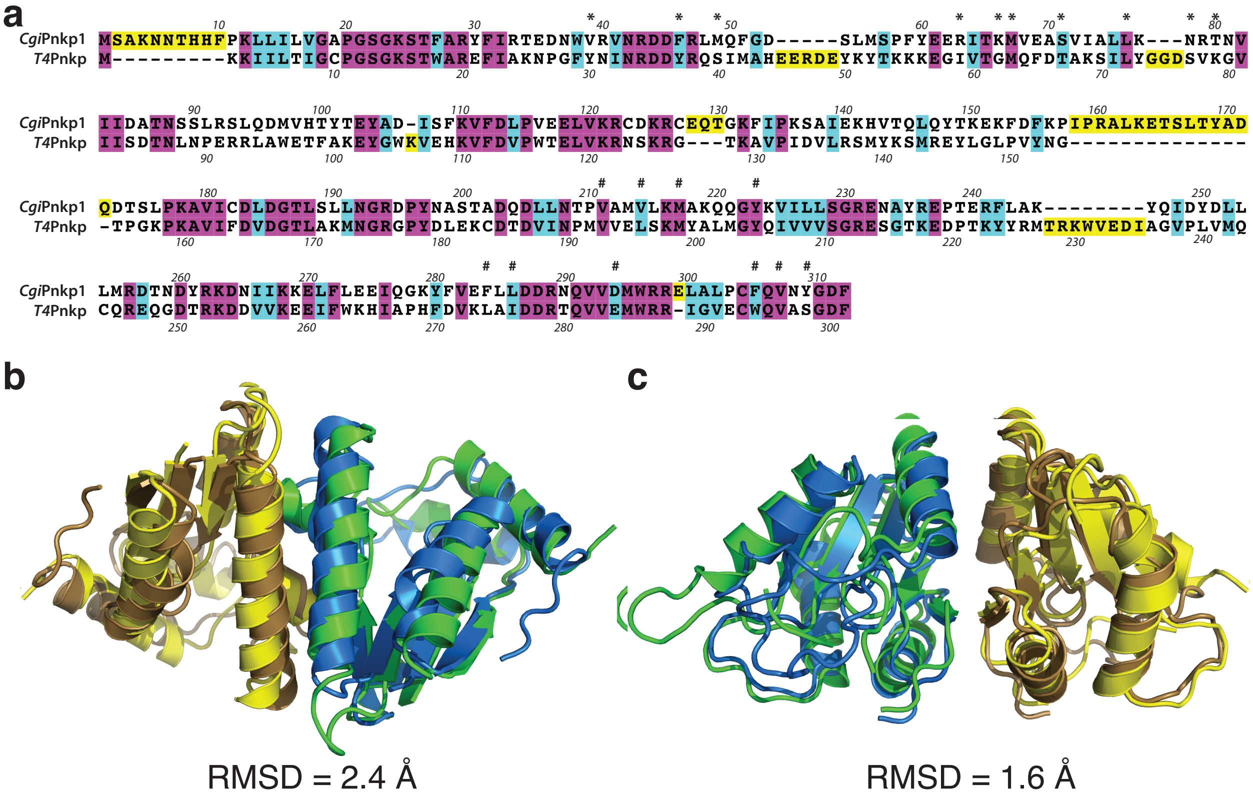

Supplementary Figure 9 | Sequence and structural comparison of *Cgi*Pnkp1 with *T4*Pnkp. (**a**) Amino acid sequences of *Cgi*Pnkp1 and *T4*Pnkp were aligned. The conserved residues are boxed in color, with completely conserved residues in magenta and similar residues in cyan. Residue numbers above the alignment belong to *Cgi*Pnkp1, and those below correspond to *T4*Pnkp. The residues marked with asterisks and number signs are involved in dimerization of kinase and phosphatase domains of *Cgi*Pnkp1, respectively. (**b**) Structural alignment of the kinase module of *Cgi*Pnkp1 (residues 2-157) with the corresponding domains in *T4*Pnkp (Accession code: 2IA5, residues 2-154)[2]. *Cgi*Pnkp1 are colored and oriented the same in Fig. 4a, and *T4*Pnkp are colored yellow and green, respectively. (**c**) Structural alignment of the phosphatase domains of *Cgi*Pnkp (residues 173-312) with the corresponding domains in *T4*Pnkp (Accession code: 2IA5, residues 155-301). The structures are colored the same as in **b** and oriented the same as in Fig. 4b.

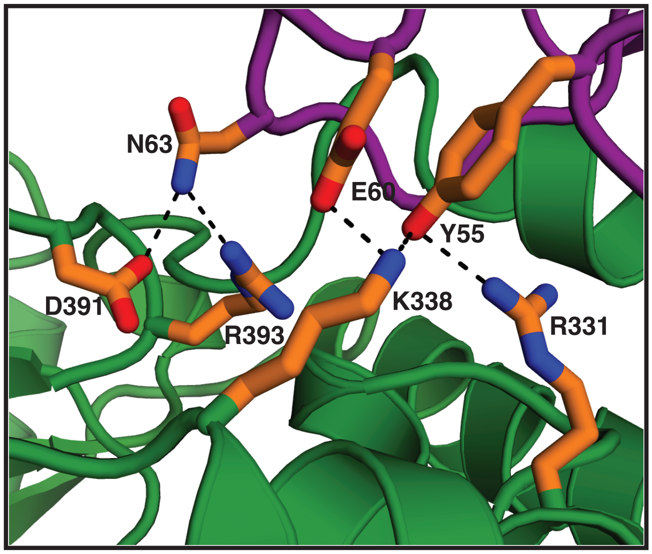

Supplementary Figure 10 | Additional interactions between Hen1 and Rnl based on the structure of Pnkp1–Rnl–Hen1 heterohexamer. In addition to the interactions shown in Fig. 4d, the side chains of three residues (Y55, E60, and N63) from a loop of the N-terminal ligase-activating domain of Hen1 form five hydrogen bonds with the side chains of four residues (R331, K338, D391, and R393) from the C-terminal part of Rnl.

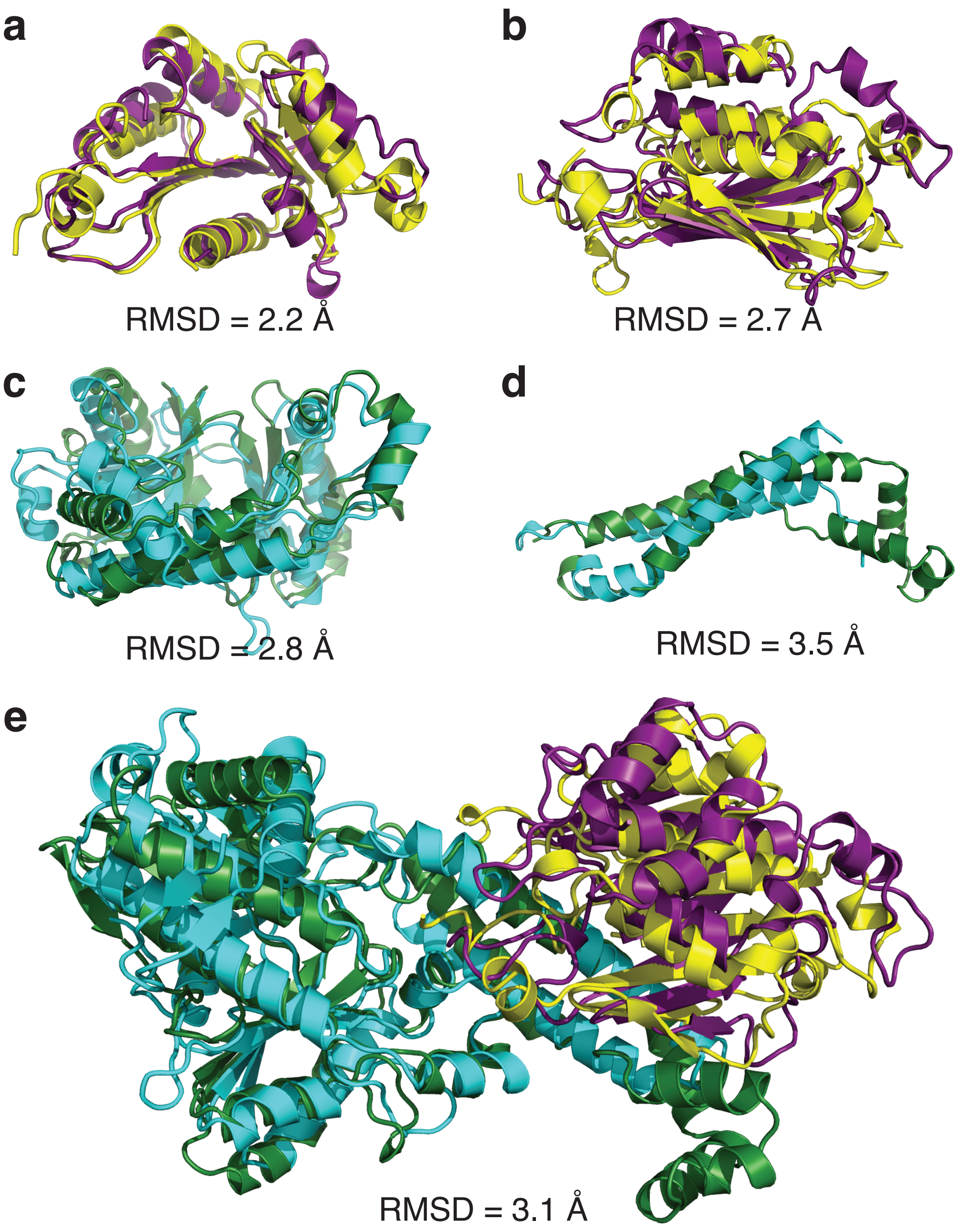

Supplementary Figure 11 | Structural similarity and difference between the Pnkp1–Rnl–Hen1 and Pnkp–Hen1 RNA repair complexes. (**a**-**e**) Structural alignments of the C-terminal methyltransferase domains of Hen1 (**a**), the N-terminal ligase-activating domains of Hen1 (**b**), ligase domains of Rnl and Pnkp (minus the insertion domains) (**c**), the insertion domains (**d**), and the entire ligase modules of Rnl–Hen1-N and Pnkp-C–Hen1-N (**e**). The structural components of the Pnkp1–Rnl–Hen1 system are depicted and colored the same as in Fig. 3a,b, and those from the Pnkp–Hen1 system are colored cyan (Pnkp) and yellow (Hen1), respectively. The comparisons are based on the structure of the C-terminal methyltransferase domain of Hen1 from *Clostridium Thermocellum* (*Cth*) (Accession code: 3JWI)[3], and the structure of the ligase module of *Cth*Pnkp–Hen1 (Accession code: 4E6N)[4].

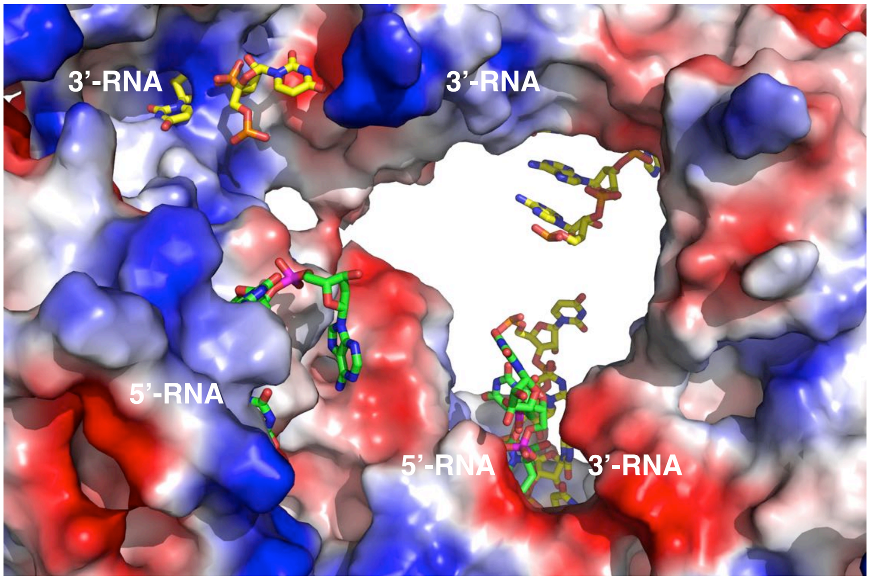

Supplementary Figure 12 | Docking single-stranded RNAs into the active sites of the Pnkp1–Rnl–Hen1 heterohexamer. The image displayed here has the same view as the one shown in Fig. 6a, but the color schemes are different. The surface of the proteins is colored with local electrostatic potential (red, negatively charged; blue, positively charged; white, neutral). The heteroatoms of the docked RNAs are colored individually, with nitrogen in blue, oxygen in red, and phosphate in magenta. The carbon atoms for the 5’-half RNA are colored green, and the ones for the 3’-half are colored yellow.

Supplementary Table 1 | List of primers used for cloning three genes to overexpress the recombinant proteins for the reconstitution of the RNA repair complex.

**Name**	DNA Sequence
Pnkp1-5’	5’-GCGTACTCTAGAAATAATTTTGTTTAACTTTAAGAAGGAGATATA CCATGAGTGCAAAAAATAATACACATC-3’
Pnkp1-3’	5’- GCGATCTGCAG TTAGAAATCACCATAGTTTACTTGAA-3’
Rnl-5’	5’-GCGTACTCTAGAAATAATTTTGTTTAACTTTAAGAAGGAGATATA CCATGGAAGATAAAACATTGATAAAAAAAC-3’
Rnl-3’	5’- GCGATCTGCAG TCATAATCTGCTATCTAATTGATTTTC-3’
Hen1-5’	5’- GCGATCATATG ATTTTACAAATACACTCCCAG-3’
Hen1-3’	5’- GCGATGGTACCTTATTTCCGAGTGATAACAACCG-3’

Supplementary references

1 Chan, C. M., Zhou, C. & Huang, R. H. Reconstituting bacterial RNA repair and modification in vitro. *Science* **326**, 247 (2009).

2 Zhu, H., Smith, P., Wang, L. K. & Shuman, S. Structure-function analysis of the 3' phosphatase component of T4 polynucleotide kinase/phosphatase. *Virology* **366**, 126-136 (2007).

3 Chan, C., Zhou, C., Brunzelle, J. S. & Huang, R. H. Structural and biochemical insights into 2'-O-methylation at the 3'-terminal nucleotide of RNA by Hen1. *Proc. Natl. Acad. Sci. U.S.A.* **106**, 17699-17704 (2009).

4 Wang, P. *et al.* Molecular basis of bacterial protein Hen1 activating the ligase activity of bacterial protein Pnkp for RNA repair. *Proc Natl Acad Sci U S A* **109**, 13248-13253 (2012).
